# Effects of environmental and social facilitators on physical activity among older adults: A population-based study in São Paulo, Brazil

**DOI:** 10.1016/j.clinsp.2026.101066

**Published:** 2026-07-23

**Authors:** Bruno Holanda Ferreira, Emerson Sebastião, Otavio Amaral de Andrade Leao, Tatiane Kosimenko Ferrari Figueiredo, Camila Nascimento Monteiro, Chester Luiz Galvão Cesar, Moisés Goldbaum, Olinda do Carmo Luiz

**Affiliations:** a Faculdade de Medicina, Universidade de São Paulo, São Paulo, SP, Brazil; b University of Illinois Urbana-Champaign, Champaign, USA; c Faculdade de Saude Pública, Universidade de São Paulo, São Paulo, SP, Brazil

**Keywords:** Urban health, Health surveys, Health inequalities

## Abstract

**Introduction:**

Regular physical activity is essential for healthy aging, but adherence among older adults remains low. Few studies have examined the influence of environmental and social factors on activity.

**Objective:**

To analyze the associations between facilitators ‒ built environment, safety, social support, and public programs ‒ and physical activity among older adults. Method: Data from 1,017 individuals aged 60-years or older from the 2015 São Paulo Health Survey were analyzed. Physical activity (≥150 min/week) was assessed using the IPAQ. Poisson regression estimated associations with individual facilitators and a cumulative score.

**Results:**

Overall, 30.1% achieved ≥150 min/week of physical activity. Awareness of public programs was associated with a higher likelihood of physical activity (PR = 1.63; 95% CI 1.28–2.09). Older adults living in the Southeast (PR = 1.55; 95% CI 1.09–2.20) and East zones (PR = 1.54; 95% CI 1.04–2.27) were more likely to achieve ≥150 min/week than those living in the North. Compared with those reporting no facilitators, the likelihood of meeting recommendations increased with the number of facilitators: PR = 1.48 (95% CI 1.00–2.19) for three facilitators, PR = 2.19 (95% CI 1.44–3.32) for four, and PR = 2.39 (95% CI 1.19–4.78) for five facilitators.

**Conclusion:**

Physical activity among older adults was associated with awareness of public programs and area of residence, suggesting territorial differences in opportunities for physical activity across São Paulo. Additionally, a cumulative pattern was observed, suggesting that the combined presence of environmental and social facilitators may further increase engagement in physical activity.

## Highlights

• Physical activity levels were low among older adults in São Paulo.
• Public program awareness was associated with higher physical activity.
• Physical activity was higher in Southeast and East than in North São Paulo.
• Proximity to public spaces and perceived safety showed no independent association after adjustment for personal and regional factors.
• A higher number of facilitators was associated with higher physical activity.

## Introduction

Regular physical activity plays a key role in healthy aging, contributing to the prevention of cardiovascular and metabolic diseases, the reduction of functional limitations, and the improvement of overall quality of life.^1^ Despite these well-established benefits, a substantial proportion of older adults do not meet current physical activity recommendations worldwide. Physical inactivity is also associated with significant economic and social burdens,^2^ including increased healthcare costs and higher mortality from non-communicable diseases.^2^, ^3^, ^4^, ^5^ Globally, 27.5% of the global population1 and 47% of the Brazilian population^1^, ^5^ do not meet current physical activity guidelines.

Understanding the factors that influence engagement in physical activity among older adults is therefore essential for developing effective public health strategies. This knowledge can support the design of targeted interventions and policies aimed at increasing physical activity levels in this population.^6^^,^^7^ Previous research has identified several determinants of physical activity, including individual characteristics, environmental conditions, access to recreational spaces, neighborhood safety, and social support.^8^, ^9^, ^10^, ^11^

Although the benefits and barriers to physical activity among older adults are well documented, there is limited evidence that integrates multiple facilitators in large urban settings. In the municipality of São Paulo, characterized by marked social and territorial inequalities, it is particularly relevant to examine how the built environment, neighborhood safety, social support, knowledge of public programs, and area of residence jointly influence physical activity among older adults. Understanding these relationships is essential for informing effective public policies and fostering more inclusive, healthier urban environments that support active aging. Thus, this study analyzed the associations between facilitators ‒ built environment, safety, social support, and public programs ‒ and physical activity among older adults residing in São Paulo, using data from the São Paulo Health Survey (ISA-Capital).

Changes in the built environment, such as the creation or improvement of public spaces that facilitate physical activity, usually occur gradually and depend on political planning, long-term investments, and intersectoral programs.^12^^,^^13^ Therefore, examining these associations using ISA-Capital 2015 provides an important baseline for understanding environmental and social facilitators of physical activity among older adults in São Paulo.

## Materials and methods

This cross-sectional, population-based, analytical study was conducted in accordance with the Strengthening the Reporting of Observational Studies in Epidemiology (STROBE) Statement. The study sample refers to the population residing in the urban area of the municipality of São Paulo. It is the largest city in the country, the main economic center, and the fourth-most populous metropolis in the world. According to estimates from the National Demographic Census during the data collection period, the municipality had approximately 11.2 million inhabitants, of whom about 11.9% were older adults.^14^

The ISA-Capital aims to assess the health status of the municipal population, considering living conditions and addressing lifestyle indicators and chronic diseases. Health information was obtained using a complex sampling design that probabilistically selected census tracts and households. Two domains were considered: geographic and demographic, encompassing the health districts (Central-West, East, North, Southeast, and South), age groups, and sex (male and female older adults), respectively. The total sample consisted of 980 older adults, with planned interviews ranging from 162 to 234 per regional coordinator. This design allowed estimation of proportions of 0.50, with a sampling error of 0.10, a 95% confidence level, and a design effect of 1.5. All selected households were visited at least three times, and the household response rate was 0.76. Considering the eligible individuals contacted in these households, the individual response rate was 0.74.^15^

The ISA-Capital 2015 survey interviewed 1,019 individuals aged 60-years or older residing in permanent private households in São Paulo. For the present analysis, bedridden individuals, those with severe mobility limitations, institutionalized residents, people experiencing homelessness, and participants with incomplete physical activity data, 1,017 individuals were included in the final analysis. After applying sampling weights, the analytical sample represented 1,602,717 older adults living in the municipality.

Data were collected using standardized questionnaires administered by trained interviewers and completed directly by older adult residents. The questionnaire was organized into thematic blocks, with most questions being closed-ended and having predefined response options. Further details on the ISA-Capital sampling design are available elsewhere.^15^

All participants signed an informed consent form and received detailed explanations about the purpose and procedures of the survey. The study complies with Resolution n° 466/12 of the Brazilian National Health Council (CNS). Ethical approval was obtained from the Research Ethics Committee of the School of Public Health, University of São Paulo, São Paulo (SP), Brazil (protocol: 719.661/2014).

### Variables

#### Physical activity

Physical activity was analyzed based on the total sum of activities performed in the leisure-time and commuting domains.^16^ Data were collected using the long version of the International Physical Activity Questionnaire (IPAQ), which has been validated for the Brazilian population^17^ and widely used in other studies based on ISA-Capital data.^16^^,^^18^^,^^19^ Questions addressed weekly frequency and total daily duration of physical activity, including items such as: “During a typical week, on how many days did you engage in vigorous physical activity for at least 10 min?” Responses ranged from 1 to 7 days. Another item asked: “How much time do you usually spend performing vigorous physical activity on one of those days?

Scores were converted to minutes per week according to the international IPAQ guidelines (www.ipaq.ki.se). To align with international physical activity recommendations, total physical activity time was calculated as: total minutes = moderate-intensity minutes + (2× vigorous-intensity minutes). Thus, minutes of vigorous-intensity activity were weighted as double, reflecting the equivalence between 150 min of moderate-intensity activity and 75 min of vigorous-intensity activity recommended by current guidelines.^20^ For analysis, total physical activity time was categorized into two groups: 0–149 min/week and ≥150 min/week, following physical activity guidelines^5^ and previous studies.^16^^,^^19^

#### Personal factors

Self-reported personal factors used in the present study included: age group (60–69, 70–79, 80 or more), sex (male, female), education level (never attended school; elementary school I; elementary school II; high school; higher education or more), and marital status (with partner, without partner).

Race/skin color was self-reported according to the categories used in the Brazilian National Census conducted by the Brazilian Institute of Geography and Statistics (IBGE): white, black, brown, yellow, and indigenous. For analytical purposes, categories were grouped due to the small number of participants in some groups. Therefore, race/skin color was categorized into three broader groups: white/yellow, black/brown/indigenous, and other. Previous analyses using population-based data in Brazil have indicated that individuals classified as “yellow” tend to present socioeconomic characteristics more similar to those of white participants than to other racial groups.^21^

For self-reported chronic conditions, the following were considered: hypertension, diabetes, angina, myocardial infarction, cardiac arrhythmia, other heart disease, cancer, arthritis, rheumatism, osteoarthritis, osteoporosis, asthma or bronchial asthma, emphysema, chronic bronchitis or chronic obstructive pulmonary disease, rhinitis, chronic sinusitis, other lung diseases, tendinitis, repetitive strain injury, work-related musculoskeletal disorder, lower-limb varicose veins, stroke or cerebrovascular accident, other vein, artery, or circulatory disease, high cholesterol, spinal disease or back problem, emotional or mental problems such as anxiety, depression, panic disorder, obsessive-compulsive disorder, schizophrenia, or others, and any other chronic disease not previously listed. Each condition was investigated using the question: “Has any physician ever told you that you have [name of the disease]?” For mental health problems, the following question was used: “Do you have any emotional or mental problem, such as anxiety, depression, panic disorder, Obsessive-Compulsive Disorder (OCD), schizophrenia, or any other?” For data analysis, the number of chronic conditions was categorized as 0–1, 2, 3, 4, 5 or more.

#### Built environment and neighborhood safety

For variables related to the built environment and neighborhood safety, the following questions were used: “Are there suitable public spaces (such as squares, parks, or streets) near your residence for engaging in physical activity?” (Proximity to public spaces: yes/no); “During the day or at night, do you feel safe walking or practicing sports near your home?” (Daytime/nighttime safety: yes/no); and “Are you aware of any public programs in your municipality that promote physical activity?” (Public programs: yes/no).

#### Social influence

Social influence was assessed using the following question: “Does any friend, relative, or neighbor invite you to walk or engage in sports in your neighborhood?” (Invitation to walk: yes/no).

#### Area of residence

The area of residence was defined based on the regional health coordinators selected in the sample, including the North, Central-West, Southeast, South, and East regions of São Paulo.

#### Facilitators for physical activity

A variable “Facilitators for physical activity” was created, with a score ranging from zero (no facilitators) to five (all reported facilitators – summing factors related to the built environment, neighborhood safety, and social influence) (yes = 1; no = 0). For example, a participant reporting all five items would have: proximity to public spaces (1); feeling safe walking or practicing sports near home during the day (1); feeling safe walking or practicing sports near home at night (1); awareness of a public program promoting physical activity (1); and receiving invitations to walk (1).

The construction of this score was conceptually informed by the Socio-Ecological Model, which emphasizes that physical activity behavior is shaped by interacting with environmental and social determinants operating at multiple levels.^7^^,^^22^ The conceptual rationale for combining these factors lies in evidence that social and environmental determinants of physical activity often exert cumulative and synergistic effects rather than acting independently.^23^ The score was therefore used as a proxy for combined exposure to favorable neighborhood conditions that may facilitate physical activity.

Although all components were treated with equal weight, the individual facilitators were also analyzed separately in the regression models to examine their independent associations with the outcome. Previous studies have shown that the simultaneous presence of multiple facilitators ‒ such as access to safe public spaces, social support, and awareness of programs ‒ tends to increase the likelihood of engagement in physical activity more strongly than any single factor alone.^7^^,^^9^^,^^24^ Pairwise correlations among the five facilitator items ranged from 0.05 to 0.27, indicating that they captured distinct but complementary dimensions of environmental and social support for physical activity. Thus, the score was intended to capture the cumulative influence of these elements within the urban context of São Paulo, where disparities in infrastructure and social resources are pronounced.

### Statistical analysis

In the descriptive analysis, variables related to personal factors, built environment and neighborhood safety, social influence, facilitators for physical activity, area of residence, and physical activity were estimated for the total sample using prevalence and their respective 95% Confidence Intervals (95% CI). To assess the association between physical activity facilitators and physical activity engagement, the Chi-Square test for trend was used. Incomplete information was identified and treated as missing. Regression analyses were conducted using complete-case analysis, including only participants with available data for all variables included in each model.

To identify potential associations between the built environment, neighborhood safety, social influence, area of residence, and number of facilitators with physical activity, Poisson regression was performed to estimate crude and adjusted Prevalence Ratios (PR) (adjusted for personal factors^24^^,^^25^ and area of residence) and their 95% CI. Poisson regression was chosen due to the study design and the high prevalence of the binary outcome (>10%), providing prevalence estimates with more conservative confidence intervals.^26^ Additionally, a sensitivity analysis was conducted by reconstructing the facilitator score without the item on public program awareness to assess the robustness of the associations and reduce the possibility of reverse causality. Both the five- and four-item scores were analyzed as categorical variables and as continuous trends (per additional facilitator) under the same adjusted Poisson regression framework.

The statistical software Stata (version 14, StataCorp, College Station, Texas, USA) was used; the *svyset* command incorporated sampling weights, primary sampling units, and strata according to the complex survey design of ISA-Capital. A significance level of 5% was adopted for all analyses.

## Results

Descriptive data for the total sample related to personal factors, built environment and neighborhood safety, social influence, facilitators for physical activity, area of residence, and physical activity are presented in detail in Table 1. Regarding personal factors, the most prevalent group included individuals aged 60–69 years, females, individuals with low educational attainment, individuals who self-identify as white or yellow to race/skin color, and those reporting 0–1 chronic condition. Regarding variables related to the built environment, neighborhood safety, and social influence, 32.1% of older adults reported feeling unsafe engaging in physical activity near their residence during the day, increasing to 82.5% at night. Awareness of public programs that offer physical activity was 22.4%, and 21% reported receiving invitations to walk.

**Table 1:** Proportion of personal factors, built environment, neighborhood safety, social influence, facilitators for physical activity, area of residence, and physical activity. ISA-Capital 2015.

Variables and Categories		Total sample	
		N^a^	% (95% CI)^b^
Personal factors	Age range (years)		
	60–69	572	57.2 (52.9–61.5)
	70–79	305	28.6 (24.7–32.4)
	80 or more	140	14.2 (11.4–17.0)
	Sex		
	Female	630	59.6 (56.8–62.4)
	Male	387	40.4 (37.6–43.2)
	Education		
	Never attended school	90	6.9 (5.1–8.7)
	Elementary School I	457	40.7 (36.2–45.3)
	Elementary School II	153	15.1 (12.7–17.5)
	High School	172	17.9 (15.4–20.3)
	Higher Education or above	139	19.4 (14.7–24.1)
	Marital status		
	With partner	515	52.9 (48.6–57.1)
	Without partner	498	47.1 (42.9–51.4)
	Race/skin color		
	White and yellow	643	68.2 (63.7–72.7)
	Black and brown	319	27.3 (22.9–31.7)
	Others	55	4.5 (3.0–6.0)
	Chronic conditions		
	0‒1	338	32.4 (28.9–35.8)
	2	192	19.2 (16.7–21.8)
	3	158	16.5 (13.9–19.2)
	4	138	12.9 (10.5–15.4)
	5 or more	191	18.9 (15.9–21.9)
Built environment and Neighborhood safety	Proximity to public spaces		
	No	441	44.9 (39.5–50.2)
	Yes	521	55.1 (49.8–60.5)
	Safe at day		
	No	323	32.1 (27.3–36.9)
	Yes	655	67.9 (63.1–72.7)
	Safe at night		
	No	804	82.5 (79.7–85.4)
	Yes	166	17.5 (14.6–20.3)
	Public programs		
	No	761	77.6 (73.9–81.2)
	Yes	233	22.4 (18.8–26.1)
Social influence	Invitation to walk		
	No	789	79.0 (76.0–81.9)
	Yes	217	21.0 (18.1–24.0)
Facilitators for Physical Activity			
0		191	18.5 (14.6–22.4)
1		252	23.9 (20.6–27.3)
2		278	29.1 (24.6–33.6)
3		211	20.1 (16.9–23.4)
4		74	7.5 (5.5–9.4)
5		11	1.0 (0.3–1.5)
Area of Residence			
North		187	16.6 (13.0–20.3)
Central-West		201	22.1 (15.8–28.3)
Southeast		260	30.4 (24.5–36.4)
South		198	18.1 (14.4–21.7)
East		171	12.8 (10.0–15.6)
Physical activity			
0–149 min/week		719	69.9 (65.8–73.9)
≥150 min/week		298	30.1 (26.1–34.2)

a N-unweighted.

b Weighted prevalence.

Regarding the set of facilitators for physical activity, most participants reported between 1‒3 facilitators, and a higher prevalence among residents in the Southeast region. The prevalence of participants reporting ≥150 min/week of physical activity was 30.1%, as shown in Table 1.

The distribution and regression analysis of time spent in physical activity among older adults, by built environment and safety, social influence, area of residence, and the number of facilitators for physical activity, are presented in detail in Table 2. When stratifying by specific facilitators, awareness of public programs promoting physical activity was associated with a 63% higher likelihood of engaging in ≥150 min/week of physical activity (PR = 1.63; 95% CI: 1.28–2.09) compared with older adults who were not aware of any public physical activity programs. Regarding area of residence, older adults living in the Southeast (PR = 1.55; 95% CI: 1.09–2.20) and East regions (PR = 1.54; 95% CI: 1.04–2.27) had a higher probability of engaging in physical activity than those living in the North region.

**Table 2:** Prevalence and Poisson regression analyses of physical activity among older adults according to built environment, neighborhood safety, social influence, area of residence, and facilitator scores. ISA-Capital 2015.

Variables and Categories		≥150 min/week		Crude model PR (95% CI)	Primary analysis (5-item score) PR (95% CI)^c^	Sensitivity analysis (4-item score) PR (95% CI)^d^
		N^a^	% (95% CI)^b^			
Built environment and Neighborhood safety	Proximity to public spaces					
	No	114	26.9 (22.5–31.9)	1	1	
	Yes	177	34.5 (28.5–41.0)	1.28 (1.00–1.63)	1.20 (0.94–1.53)	
	Safe at day					
	No	75	25.7 (20.0–32.3)	1	1	
	Yes	217	33.0 (28.2–38.2)	1.29 (0.97–1.70)	1.22 (0.92–1.61)	
	Safe at night					
	No	224	29.0 (24.8–33.5)	1	1	
	Yes	61	36.0 (27.4–45.7)	1.24 (0.93–1.66)	1.21 (0.92–1.58)	
	Public programs					
	No	196	26.5 (22.2–31.2)	1	1	
	Yes	100	44.9 (37.4–52.6)	1.70 (1.35–2.14)	1.63 (1.28–2.09)	
Social influence	Invitation to walk					
	No	221	28.9 (24.9–33.2)	1	1	
	Yes	77	36.6 (29.3–44.5)	1.27 (1.02–1.58)	1.22 (0.99–1.49)	
Area of Residence	North	42	21.9 (15.9–29.3)	1	1	
	Central-West	70	33.2 (22.4–46.1)	1.52 (0.95–2.44)	1.39 (0.88–2.19)	
	Southeast	80	33.6 (26.7–41.1)	1.53 (1.05–2.23)	1.55 (1.09–2.20)	
	South	49	25.7 (19.5–32.9)	1.17 (0.78–1.76)	1.21 (0.83–1.77)	
	East	57	33.8 (25.3–43.6)	1.55 (1.03–2.34)	1.54 (1.04–2.27)	
Number of facilitators for physical activity (5-item score)	0	34	20.4 (14.1–28.5)	1	1	1
	1	67	25.2 (20.0–31.2)	1.23 (0.81–1.89)	1.15 (0.77–1.71)	1.25 (0.87–1.79)
	2	81	31.3 (24.3–39.2)	1.53 (1.02–2.31)	1.44 (0.96–2.15)	1.36 (0.96–1.94)
	3	69	33.9 (26.9–41.7)	1.67 (1.10–2.51)	1.48 (1.00–2.19)	1.44 (1.00–2.07)
	4	39	51.9 (37.6–65.9)	2.55 (1.67–3.88)	2.19 (1.44–3.32)	2.21 (1.46–3.36)
	5	8	61.7 (26.7–87.8)	3.03 (1.55–5.93)	2.39 (1.19–4.78)	–
	Per 1 facilitator (continuous)			1.19 (1.10–1.29)	1.15 (1.05–1.26)	

a N unweighted

b Weighted prevalence estimates presented; Bold values indicate statistical significance (*p* < 0.05).

c Adjusted for personal factors and area of residence using the 5-item facilitator score.

d Sensitivity analysis using the 4-item facilitator score, excluding awareness of public physical activity programs.

A dose–response gradient was identified between the number of facilitators and engaging in ≥150 min/week of physical activity (*p*-trend < 0.001). In the primary analysis using the five-item score, a dose-response pattern was observed, with associations becoming evident from three facilitators onwards; however, the estimate for three facilitators was borderline statistically significant (PR = 1.48; 95% CI: 1.00–2.19). Associations were also observed for four facilitators (PR = 2.19; 95% CI: 1.44–3.32) and five facilitators (PR = 2.39; 95% CI: 1.19–4.78). In the sensitivity analysis excluding awareness of public programs (four-item score), the dose–response pattern remained consistent, although influenced by the composition of its components and became evident at lower levels of exposure: borderline associations were already observed at two facilitators (PR = 1.36; 95% CI: 0.96–1.94) and three facilitators (PR = 1.44; 95% CI: 1.00–2.07), reaching PR = 2.21 (95% CI: 1.46–3.36) for four facilitators. In continuous models, each additional facilitator increased the likelihood of meeting the activity recommendations by 19% for the five-item score and by 15% for the four-item score (Table 2).

**Fig. 1:**
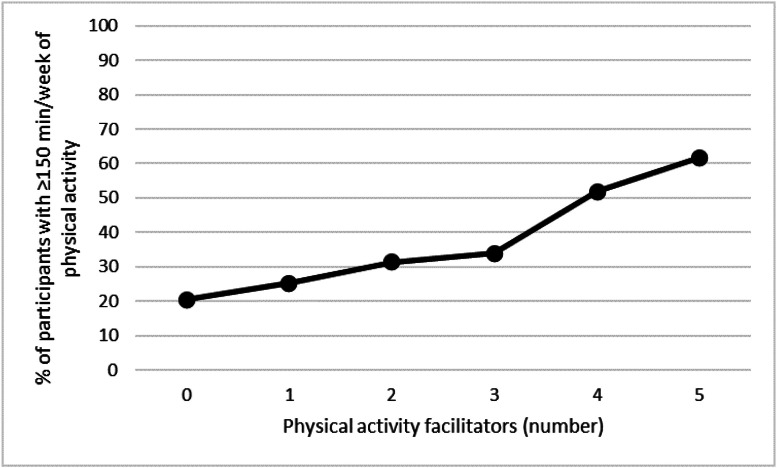
Weighted prevalence of older adults achieving ≥150 min/week of physical activity according to the number of facilitators (ISA-Capital 2015); Chi-Square test for trend (<0.001).

Fig. 1 shows a positive trend between the number of facilitators for physical activity and the higher prevalence of engaging in ≥150 min/week of physical activity (*p*-trend < 0.001). It can be observed that as the number of facilitators increases, the proportion of individuals who practice ≥150 min/week of physical activity rises progressively: 20.4%, 25.2%, 31.3%, 33.9%, 51.9%, and 61.7%.

## Discussion

This study analyzed the association between factors related to the built environment, neighborhood safety, social support, and public programs with physical activity in a representative sample of older adults living in the city of São Paulo. The results showed that 30.1% of participants reported engaging in ≥150 min/week of physical activity. When individual facilitators were examined, awareness of public programs promoting physical activity showed the strongest association with meeting physical activity recommendations. At the same time, residents of the Southeast and East zones had a significantly higher likelihood of engaging in physical activity than residents of the North zone. When the cumulative number of facilitators was analyzed, a dose–response gradient was observed between the number of facilitators and meeting physical activity recommendations.

Brazilian studies have monitored in recent decades the prevalence of physical activity among older adults, consistently reporting low levels and a stable trend over time.^6^ In the present study, only 30.1% of participants reported engaging in ≥150 min per week of physical activity, a proportion that, although concerning, is higher than that reported in some areas of Brazil, such as a Northeastern state, where prevalence reached only 12.5%.^27^ On the other hand, the results were lower than those observed in a Southern region of the country, where prevalence was 46% among women and 60.4% among men.^28^ Still, the findings of the present study are consistent with another investigation conducted in the city of São Paulo,^16^^,^^19^ and reinforce the worrisome low prevalence, even before the COVID-19 pandemic. However, comparisons of prevalence estimates across studies should be interpreted with caution, as differences in measurement instruments, domains of physical activity assessed, operational definitions of physical activity, and sampling designs may substantially influence the reported prevalence. International guidelines recommend that older adults accumulate at least 150 min per week of moderate-intensity physical activity. When this is not possible, individuals are encouraged to remain as physically active as possible and gradually increase activity levels according to their functional capacity and health status.^29^

Among the factors analyzed individually, knowledge of public physical activity programs stood out, increasing the likelihood of identifying individuals engaging in ≥150 min/week of physical activity by 63%, even after adjusting for personal and regional factors. This finding reinforces the relevance of disseminating information and ensuring accessible opportunities for regular practice, particularly through government initiatives such as the “Vida Ativa” and “Academia da Saúde” programs, which have already been documented as effective in the literature.^5^^,^^11^ International evidence supports this result: a systematic review found that most social marketing interventions targeting older adults led to an increase in physical activity, underscoring the importance of information dissemination and communication strategies tailored to this age group.^30^ The low awareness of public physical activity programs observed in this study (22.4%) also suggests a substantial gap between the availability of community-based initiatives and older adults’ knowledge of these opportunities, representing a potentially modifiable target for public health action.

The area of residence also significantly influenced the time spent on physical activity. Older adults living in the Southeast and East zones had a higher probability of engaging in ≥150 min/week of physical activity than those living in the North zone, possibly reflecting regional inequalities in infrastructure, availability of public facilities, or community-based health promotion initiatives. These territorial disparities, previously observed in another urban survey^31^ reinforce the need for public policies that ensure a more equitable spatial distribution of resources.

Variables such as the presence of nearby public spaces and perceptions of neighborhood safety, although positively associated in the crude model, did not remain significant after adjustment. In contrast, Monteiro et al., in Criciúma (SC), observed that public places for exercise, sidewalks, and pedestrian crossings were positively associated with leisure-time and active transportation physical activity.^32^ This discrepancy may reflect contextual differences between large capitals, such as São Paulo, and medium-sized cities. It is also possible that in a megacity such as São Paulo, the high density and heterogeneity of public spaces may dilute the effect of their mere presence, making factors such as quality, maintenance, accessibility, and usability more relevant for promoting physical activity among older adults. The lack of association observed for proximity to public spaces and perceived neighborhood safety after adjustment may partly reflect limitations in how these variables were measured in the survey. Proximity to public spaces was assessed using a dichotomous self-reported question, which does not capture important attributes such as distance, accessibility, infrastructure quality, maintenance, aesthetics, or the availability of facilities appropriate for older adults. Previous research suggests that these environmental characteristics may play a more decisive role in influencing physical activity than the mere presence of public spaces. Review studies have emphasized that the mere existence of infrastructure does not guarantee engagement in physical activity unless it is accompanied by conditions of accessibility, attractiveness, and perceived safety.^9^^,^^11^ Findings from a systematic review further indicated that, at the community level, environments featuring parks, fitness centers, and adequate facilities tend to facilitate regular physical activity.^24^

Social influence, as reflected in invitations from friends, relatives, or neighbors to go for a walk, was not significantly associated with physical activity in the adjusted model. The literature recognizes social support as an essential determinant of physical activity among older adults, particularly for its potential to foster motivation and engagement.^8^^,^^10^^,^^13^^,^^24^^,^^33^ However, the findings of this study did not confirm this association. One possible explanation is that the measure used captures only a limited dimension of social support. Social support may also operate through informational pathways, such as encouragement to participate in organized community programs or group-based activities. In this context, awareness of public programs, which showed a strong independent association with physical activity, may partially overlap with or mediate certain forms of social support. However, the available data do not allow a more detailed examination of these potential interactions. Future studies should investigate how different dimensions of social support interact with community-based programs to influence physical activity among older adults.

Patel et al. have indicated that the presence and involvement of family and friends can act as a facilitating factor.^33^ This differs from the findings in Criciúma (SC), where invitations from friends and relatives, as well as community sporting events, increased the likelihood of leisure-time walking.^32^ These findings suggest that the influence of social support may vary with social and environmental contexts and the types of opportunities available for physical activity.

The findings indicate a positive, gradual association between the number of facilitators and the proportion of older adults who engage in ≥150 min/week of physical activity. Similar patterns were observed in other research, where higher scores of a favorable perceived environment were associated with a greater prevalence of physical activity.^32^ These consistent findings underscore the cumulative and synergistic effect of multiple social and environmental facilitators. Those who reported multiple factors, such as accessible public spaces, neighborhood safety, social support, and awareness of public programs, were more likely to engage in at least 150 min/week. This pattern suggests that the accumulation of positive elements in the daily environment may exert a synergistic effect on the adoption of healthy behaviors.

In addition to the multiple facilitators identified in the present study, evidence suggests that strategies to increase the time spent in physical activity among older adults should consider multicomponent interventions. Research indicates that intergenerational activities, whether structured or not, involving people of different age groups, promote social interaction, mutual learning, and social support.^24^ Furthermore, individual factors such as enjoyment, perceived benefits, and personal interest in exercise play a central role in encouraging older adults to participate in regular physical activity programs.^24^

The sensitivity analysis also suggested that awareness of public programs may play a particularly influential role among the facilitators examined, highlighting the importance of information dissemination and accessibility of structured opportunities for physical activity.

This study has limitations that warrant consideration. It is also important to consider that the data were collected before the COVID-19 pandemic, which substantially altered mobility patterns and the use of public spaces in many cities. Therefore, the present findings should be interpreted as reflecting the pre-pandemic context. Nevertheless, the study provides valuable insights into structural environmental and social facilitators of physical activity that remain relevant for understanding long-term determinants of active aging.

Although the cross-sectional design precludes causal inference, the sensitivity analysis demonstrated that the association between facilitators and physical activity was not dependent on awareness of public programs, which could be prone to reverse causality (i.e., more active individuals being more aware of available programs). When this item was removed, the dose-response relationship remained evident, with significant effects already observed at intermediate levels of facilitators. Another limitation is that the facilitator score assumes equal contribution of all components and does not account for potential differences in the relative influence of individual factors. Therefore, the score should be interpreted as a measure of cumulative exposure to supportive environmental and social conditions rather than as an indication that all facilitators exert identical effects.

The use of self-reported data may be subject to recall or social desirability bias, potentially leading to an overestimation of the prevalence of healthy behaviors. Furthermore, some potentially relevant variables ‒ such as the quality of public spaces or the availability of well-maintained facilities ‒ were not included in the survey. In addition, São Paulo has experienced important urban transformations in recent years, including expansions of cycling infrastructure, changes in physical activity promotion programs, and investments in public spaces. Therefore, the present findings should be interpreted within the context of the ISA-Capital 2015 survey and may serve as a baseline for future studies examining how these urban changes influence physical activity among older adults.

Despite the limited generalizability of the findings, particularly to cities with lower population densities of older adults, the study benefits from a representative sample of older adults living in São Paulo and provides relevant evidence to guide public health promotion policies, offering important and novel insights by addressing key aspects of the urban environment and their relationship with physical activity among older adults. In addition, the generalizability of the findings to older adults with severe mobility limitations or those living in institutional settings is limited, as these groups were excluded from the study population. These findings may help inform future strategies to promote physical activity in this rapidly growing segment of the population, not only in Brazil but also worldwide.

From a public health perspective, these findings underscore the importance of multisectoral, coordinated approaches that account for the multiple social and environmental determinants of physical activity. In large, socially heterogeneous cities such as São Paulo, interventions should combine improvements to the built environment with strategies to strengthen community engagement and awareness of existing opportunities for physical activity. For example, targeted investments in areas with lower levels of physical activity, such as the North zone identified in this study, could focus on improving the accessibility, maintenance, and safety of public spaces. At the same time, strengthening community-based programs such as “Academia da Saúde” and expanding outreach through primary health care services (e.g., community health workers) may increase awareness and participation. Together, these actions may help reduce territorial inequalities and promote active aging in urban settings.

## Conclusion

This study demonstrated that physical activity among older adults in São Paulo is associated with multiple facilitators and, independently, with knowledge of public programs that promote physical activity. The relevance of awareness of public programs as a key element of older adults’ engagement is also highlighted, along with regional differences in opportunities for physical activity, underscoring the need for targeted investments in areas with lower levels of physical activity. A dose-response relationship was also observed, indicating a higher likelihood of identifying older adults engaged in ≥150 min/week of physical activity as the number of facilitators increased. This finding should be interpreted as an exploratory indication of cumulative exposure, complementing the results from individual facilitators rather than representing a single underlying construct.

Accordingly, public policies aimed at promoting physical activity among older adults should consider multicomponent and place-based approaches that expand access to safe, adequate environments, strengthen social support, and widely disseminate existing programs. Such measures are essential to foster active aging and reduce health inequalities in large Brazilian cities.

## Authors’ contributions

Bruno Holanda Ferreira: Collaborated in the design of the article; methodology; data analysis, and in the writing of the article.

Emerson Sebastião: Collaborated in data analysis; writing and review of the article.

Otavio Amaral de Andrade Leao: Collaborated in data analysis, writing, and review of the article.

Tatiane Kosimenko Ferrari Figueiredo: Collaborated in data analysis, writing, and review of the article.

Camila Nascimento Monteiro: Collaborated in data analysis, writing, and review of the article.

Chester Luiz Galvão Cesar: Collaborated in data analysis, writing, and review of the article.

Moises Goldbaum: Collaborated in monitoring the analysis; writing and review of the article.

Olinda do Carmo Luiz: Collaborated in the design of the article; monitoring of analyses; supervision; writing, review, and editing of the article.

## Funding and acknowledgements

BHF; Supported by the São Paulo Research Foundation (FAPESP), which financed the postdoctoral scholarship (grant n° 2024/02989-4); https://fapesp.br and the São Paulo Municipal Health Department for their support.

## Declaration of competing interest

The authors declare no conflicts of interest.

## Data Availability

The datasets used and/or analyzed during the current study are available from the corresponding author on reasonable request.

## Appendix Supplementary materials

Image, application 1
